# The expression levels of MicroRNA-146a, RANKL and OPG after non-surgical periodontal treatment

**DOI:** 10.1186/s12903-021-01883-8

**Published:** 2021-10-13

**Authors:** Mandana Sattari, Ramezan Ali Taheri, Reza ArefNezhad, Hossein Motedayyen

**Affiliations:** 1 Department of Immunology, Faculty of Medicine, Shahid Beheshti University of Medical Sciences, Tehran, Iran; 2 Nanobiotechnology Research Center, Baqiyatallah University of Medical Sciences, Tehran, Iran; 3 Department of Anatomy, School of Medicine, Shiraz University of Medical Sciences, Shiraz, Iran; 4 Autoimmune Diseases Research Center, Shahid Beheshti Hospital, Kashan University of Medical Sciences, 5th Kilometer of Ravand Road, Kashan, Iran

**Keywords:** MiR-146a, OPG, Periodontitis, RANKL

## Abstract

**Objective:**

MicroRNA-146a (miR-146a) is a regulator of inflammatory response. Periodontitis is a disease with immune pathophysiology of the periodontium in which the inflammation results in the destruction of the soft tissues and alveolar bone. Therefore, the aim of this study was to investigate the expressions of miR-146a, OPG, and RANKL in diseased and healthy periodontal tissues to understand whether miR-146a expression level may associate with OPG and RANKL mRNA levels and OPG/RANKL ratio after non-surgical periodontal treatment.

**Methods:**

The levels of miR-146a, RANKL, and OPG in gingival tissues from patients with generalized periodontitis stages II and III and grades A and B (n = 15, group A), patients with generalized periodontitis stages III and IV and grade C (n = 15, group B), and healthy individuals (n = 10) were determined by real-time PCR. The associations of miR-146a expression with OPG and RANKL levels were evaluated.

**Results:**

The levels of miR-146a in two subgroups within periodontitis patients were significantly higher than healthy subjects (*P* < 0.0001). MiR-146a showed the increased level in group A of patients compared with group B (*P* < 0.05). Clinical parameters such as probing depth (PD) and clinical attachment loss (CAL) were significantly higher in patients than control group (*P* < 0.05). The levels of OPG and RANKL were increased in patients compared with healthy subjects, although the elevated levels were not statistically significant. MiR-146a was not associated with OPG and RANKL levels and OPG/RANKL ratio.

**Conclusions:**

The results of this study failed to show the associations of miR-146a level with OPG and RANKL levels and OPG/RANKL ratio in periodontitis after non-surgical periodontal treatment.

## Background

MicroRNAs (miRNAs) are small ribonucleic acids (RNAs) which participate in the gene regulation at post-transcriptional level affecting more than 30% of human genes [1, 2]. They bind to the complementary regions in messenger RNAs (mRNAs) resulting in their degradation and/or translation inhibition [1]. MicroRNA-146a (miR-146a) as a member of miRNAs controls innate immunity and inflammation through down-regulation of inflammatory mediators [3, 4].

Periodontitis is a disease with immune pathophysiology of the periodontium which the inflammation damages the soft tissues and destroys the alveolar bone [5]. The consequences are detachment of gingival tissues from the teeth, destruction of alveolar bone, and loss of teeth [5].

Several studies have revealed that periodontitis development is largely related to inflammatory status and enzymatic activities [6, 7]. There are several reports pointing to the roles of IL-6 and nod-like receptor family pyrin domain-containing protein-3 (NLRP3), as a component of the innate immune system, in the pathogenesis of periodontitis [8]. Previous studies have indicated that patients with periodontitis had the increased levels of serum and salivary NLRP3 and interleukin-6 (IL-6) in comparison with healthy controls [6, 8]. In line with the impacts of enzymatic activities on disease progression, it is ied the key roles of different forms of transglutaminase in gingival remodelling/healing, and adaptive processes [7]. Currò et al., showed that the mRNA expressions of transglutaminase 1 and transglutaminase 3 were significantly decreased in periodontitis patients compared with healthy controls [7]. Regarding the role of transglutaminases in cross-linking of proteins involved in cell adhesion and stabilization of extracellular matrices [7], impaired functions of this enzyme in the damaged gingival may accelerate the development of the disease.

Alveolar bone destruction is stimulated by osteoclasts. The differentiation and function of osteoclast are induced by receptor activator of nuclear factor-kB ligand (RANKL) upon binding to its receptor, RANK [9]. Osteoprotegrin (OPG) is a decoy receptor which binds to RANKL and inhibits its binding to RANK, and thereby preventing osteoclastogenesis [9]. The inflammation affects bone homeostasis through enhancing the expression of RANKL and reducing the production of OPG [9].

Extensive data from the literature have indicated that patients with periodontitis had the increased level of miR-146a in comparison with healthy subjects [10–12]. Previous studies revealed that the level of miR-146a was directly correlated to the clinical features of disease severity including probing depth (PD) and clinical attachment loss (CAL). It is suggested that miR-146a may contribute to the pathogenesis of the disease [13, 14]. Furthermore, animal studies have provided some evidence to show that miR-146a exerts anti-inflammatory impacts in periodontitis through inhibiting the expressions of the pro-inflammatory cytokines such as tumor necrosis factor-alpha (TNF-α), interleukin-1β (IL-1β), and IL-6 in periodontal tissue [10, 15, 16]. On the other hand, several reports have demonstrated that pro-inflammatory cytokines stimulate the expression of RANKL and suppress the production of OPG [17–19]. Regarding inhibitory effects of miR-146a on pro-inflammatory cytokine productions, the critical question is whether RANKL and OPG expressions are indirectly affected by this microRNA. Our study was therefore focused on investigating the expressions of miR-146a, OPG, and RANKL in diseased and healthy periodontal tissues to understand whether miR-146a is correlated to RANKL and OPG levels and OPG/RANKL ratio after non-surgical periodontal treatment.

## Methods

### Sample collection and clinical examination

Gingival tissue samples were obtained from 30 periodontitis patients and 15 healthy subjects. Patients were divided into two groups according to the new classification of periodontitis [20] including: (1) patients with generalized periodontitis stages II and III and grades A and B (n = 15, group A); (2) patients with generalized periodontitis stages III and IV and grade C (n = 15, group B). The diagnosis of periodontitis and its stage, extent, and grade was performed by a periodontist based on the new classification of periodontitis [20]. Supra- and sub-gingival scaling, polishing, and oral hygiene instructions were performed at least 1 month before surgery as the preliminary phase of periodontal therapy. The average sizes of gingival tissue samples obtained from periodontits and healthy subjects were about 25 and 8.5 mm, respectively.

Gingival tissues used as control group were obtained from healthy individuals during crown-lengthening surgery with the inclusion criteria including PD < 3 mm, CAL < 3 mm and no evidence of the alveolar bone destruction based on the radiographic images.

The informed consent was collected from the participants. This study was approved by the Ethics Committee of Kashan University of Medical Sciences and performed according to the principles of Helsinki Declaration. Clinical parameters (PD and CAL) were measured by a calibrated periodontal probe (Hu Friedy, Chicago, Illinois, USA).

### Measurements of the expression levels of miR-146a, OPG, and RANKL

To determine the relative expression level of miR-146a, gingival tissue samples were homogenized using 1.0-mm silicon carbide beads (BioSpec products, Bartlesville, OK, USA). The researchers were blinded to sample information. Total RNA was isolated using a mirVana miRNA isolation kit (Ambion, Thermo Fisher Scientific, Waltham, MA, USA) according to the manufacturer’s protocol. The yield and purity of RNAs were quantified by nano drop (BioTek, Epoch, USA). Afterwards, RNA was reverse transcribed to complementary DNA (cDNA) using a TaqMan microRNA Reverse Transcription Kit (Applied Biosystems, Thermo Fisher Scientific, USA) based on the manufacturer’s instructions. The level of mature miR-146a was quantified by real-time polymerase chain reaction (Real-time PCR). This method was performed using a Rotor Gene 6000 (Qiagen, Hilden, Germany), TaqMan® Universal Master Mix II, no UNG, and hsa-miRNA146a kits (Applied Biosystems, USA). The machine was programmed as follows: 30 s incubation at 95 °C followed by 40 cycles consisted of a 95 °C denaturing temperature for 5 s and an annealing-extension temperature at 60 °C for 30 s. A non-template control containing 0.5 µL of Dnase/Rnase free water was used as the negative control. All analyses were performed in duplicate.

To measure the levels of OPG and RANKL, cDNAs were synthesized from total RNAs using a RevertAid First Strand cDNA Synthesis Kit (Thermo FisherScientific, Wilmington, Delaware, USA). Afterwards, Real-time PCR was performed in a reaction mixture consisted of Master Mix (7.5 µL, SYBR® Premix Ex Taq™ II, TaKaRa, Japan), 10 pM forward and reverse primers (0.5 µL), cDNA template (0.5 µL), and DNase-RNase free water (6 µL). The cycling parameters for OPG and RANKL were the same as those used to miR-146a. The melting curve was generated in 55 to 99 °C temperature range. Sequences of the primers are shown in Table 1.

Glyceraldehyde 3 phosphate dehydrogenase (GAPDH) gene expression served as endogenous control to normalize the expression of genes (Table 1). With the exception of the annealing temperature (55 °C for 30 s), the mixture reaction and cycling parameters were similar to those for RANKL and OPG. Relative expressions of OPG and RANKL were calculated using 2^−∆∆ct^ method [21].

**Table 1:** Sequences of primers used in the real-time PCR

Genes	Forward primer (5′-3′)	Reverse primer (5′-3′)
OPG	GTTTCCGGGGACCACAATGA	ACACGGTCTTCCACTTTGCT
RANKL	AGAGCAGAGAAAGCGATGGT	GATGGGATGTCGGTGGCATTA
GAPDH	CTCTGGTAAAGTGGATATTG	GGTGGAATCATATTGGAACA

RANKL: receptor activator of nuclear factor-kB ligand; OPG: osteoprotegrin; GAPDH: glyceraldehyde 3 phosphate dehydrogenase

### Statistical analysis

Data analyses were carried out using the SPSS program (v. 20; SPSS, Chicago, USA). The results are presented as mean ± standard deviation (SD). Kolmogrov-Smirnov test was used to determine normal distribution of data. The comparisons of two groups in normal and non-normal distribution cases were performed by using student’s t-test and Mann–Whitney U test, respectively. The correlation analyses were done using Pearson’s test (for normal distributions) and Spearman’s test (for non- normal distributions). *P* value < 0.05 was considered statistically significant.

## Results

### Clinical findings

To determine clinical features of patients and confirm periodontitis, the PD and CAL values of patients and healthy subjects were assessed. As shown in Table 2, patients in two subgroups within periodontitis had statistically significant higher PD and CAL scores than control group (*P* < 0.05).

**Table 2:** The demographic and clinical characteristics of patient and healthy individuals

	Group A	Group B	Healthy subjects
Age	42 ± 8	31 ± 7	29 ± 10
CAL (mm)	4.5 ± 0.35*	7 ± 0.53*	0.25 ± 0.28
PD (mm)	5.78 ± 0.44*	5.35 ± 0.41*	2.25 ± 0.25

Data are representative of the mean ± SD. The scores of CAL and PD in periodontitis patients were significantly higher than healthy subjects. *P < 0.05

### Expression level of miR-146a in gingival tissues of participants

**Fig. 1:**
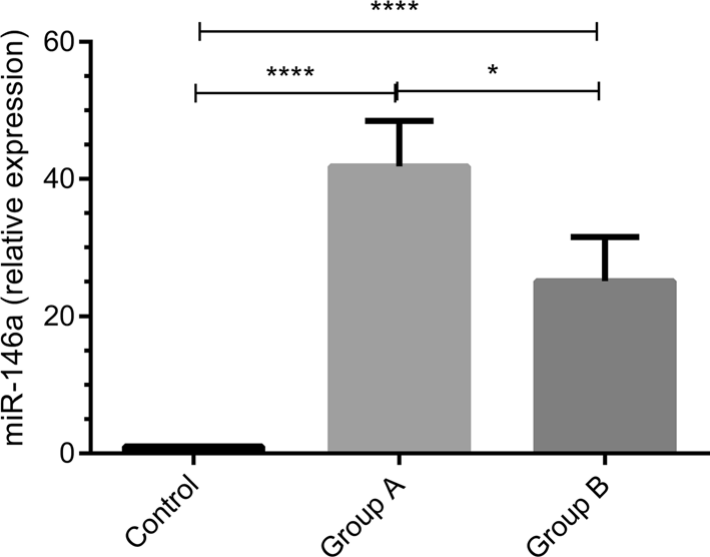
The expression levels of miR-146a in patient and healthy subjects. The levels of miR-146a in gingival tissues from group A (n = 15) and B (n = 15) of patients and healthy subjects (n = 15) were measured by real-time PCR. Data are shown as mean ± SD of relative expression of miR-146a.  *p < 0.05, ^****^ *p* < 0.0001

Regarding the fact that miR-146a can be considered as a negative feedback regulator of innate immunity and has possible role in the pathogenesis of some inflammatory diseases, the expression level of miR-146a in periodontitis was investigated. Our results revealed that both groups of patients with generalized periodontitis had an increased level of miR-146a compared with healthy individuals after one-month initial unsuccessful non-surgical treatment (P < 0.001, Fig. 1). In addition, miR-146a level was significantly higher in group A of patients (subjects with generalized periodontitis stages II and III and grades A and B) than group B of patients (subjects with generalized periodontitis stages III and IV and grade C) (*P* < 0.05, Fig. 1).

### The expression levels of RANKL and OPG in gingival tissues of participants

**Fig. 2:**
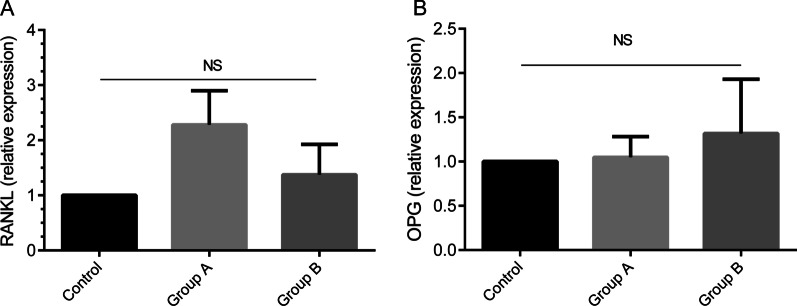
The levels of RANKL and OPG in patient and healthy subjects. The relative expressions of RANKL and OPG in group A (n = 15) and B (n = 15) of patients and healthy subjects (n = 15) were measured by real-time PCR. The results are shown as mean ± SD

Having considered that RANKL induces the destruction of alveolar bone, its expression level was analyzed using real-time PCR assay. The expression level of RANKL in gingival tissue of patients in two groups of generalized periodontitis did not statistically differ from that of healthy individuals (Fig. 2A).

In addition, the expression level of OPG, a main inhibitor of the bone destruction, in both groups of patients did not show significant changes compared with healthy subjects (Fig. 2B).

### Associations of miR-146a level with RANKL and OPG levels and OPG/RANKL ratio in gingival tissues

To explore possible mechanism(s) suggesting miR-146a impact on the pathogenesis of periodontitis, correlations of miR-146a level with RANKL and OPG were analyzed. MiR-146a level was not associated with the levels of OPG and RANKL.

In the next step, the relationship between miR-146a and OPG/RANKL ratio was evaluated. The results of spearman’s test revealed that miR-146a level was not associated with OPG/RANKL ratio.

## Discussion

MiRNAs have an important role in several biologic processes, including differentiation, proliferation, and apoptosis of the cells [22, 23]. It is reported that changes their levels participate in the development of different diseases such as cancers, cardiovascular diseases, chronic hepatitis, and diabetes [24–27]. MiR-146a controls inflammatory responses through down-regulating the expressions of IL-1 receptor-associated kinase-1 (IRAK-1) and tumor necrosis factor receptor-associated factor 6 (TRAF 6) [28, 29]. Thus, dysfunction and/ or down-regulation of miR-146a results in the pathogenesis of inflammatory diseases, especially periodontitis [11]. In our knowledge, there is no report pointing to the relationships of miR-146a with OPG and RANKL levels in periodontitis after non-surgical treatment. This study was therefore focused on determining the possible correlations of miR-146a in this filed. The results of the current study showed that miR-146a expression level is not related to OPG and RANKL levels and OPG/RANKL ratio in periodontitis patients after initial periodontal therapy.

In previous study, we observed the level of miR-146a in gingival tissues of patients with chronic periodontitis, a form of the disease according to the update of the 1999 American academy of periodontology classification criteria [30, 31], was directly correlated to clinical scores (CAL and PD) of periodontitis. Furthermore, it was observed that level of miR-146a had a direct association with the clinical scores of disease severity in aggressive periodontitis [13, 14], as another form of the disease according to the old classification of periodontitis [30]. These findings suggest that miR-146a may be associated with the pathobiology of disease. In an effort to explore possible downstream targets by which miR-146a may contribute to the pathobiology of disease, the levels of the main pro-inflammatory cytokines such as TNF-α, IL-1β, and IL-6 were studied in patients with chronic and aggressive periodontitis [13, 14]. With the expectation of IL-1β level in chronic periodontitis, the results showed significant reductions in the levels of pro-inflammatory cytokines in two subgroups within periodontitis patients [13, 14]. However, these studies failed to indicate the associations of pro-inflammatory cytokines with the major clinical factors of disease severity. Thus, the key question was how miR-146a may participate in the development and outcome of the disease. In the present study, the levels of two molecular factors involved in the pathogenesis of periodontitis along with miR-146a level were investigated.

Our study showed that miR-146a had an increased level in two subgroups within periodontitis. Interestingly, group A of patients experienced a significant increase in miR-146a level compared with group B of patients. In agreement with these observations, our previous studies revealed that the expressions of IL-1β, IL-6, and TNF-α were higher in gingival tissue of patients with chronic periodontitis than subjects with aggressive periodontitis [13, 14]. These findings suggest that inflammation reactions have higher severity in chronic periodontitis or early disease stages than aggressive form or late disease stages, which the disease progression occur in higher rate [32]. Regarding the anti-inflammatory impacts of miR-146a, it is likely that the elevated level of this miRNA is a regulatory response to control destructive and inflammatory responses, leading to the reductions in the destructions of the connective tissue and alveolar bone in group A of patients compared with subjects in group B.

In an attempt to determine the possible effects of miR-146a on other biomarkers involved in the pathogenesis of periodontitis, the levels of OPG and RANKL were assessed in two groups of patients. The elevated expressions of OPG and RANKL were observed in periodontitis patients upon preliminary phase of periodontal therapy, although these increases were not statistically significant, due perhaps to low sample size and possible effects of non-surgical periodontal treatment. Furthermore, the expression levels of RANKL and OPG were respectively decreased and increased upon disease development. These findings were in contrast with the results of some studies indicating the elevated level of RANKL accompanied by a significant reduction in OPG level during disease progression [33–35]. However, immunohistochemical studies on periodontal tissues have revealed a negative expression of RANKL and a positive expression of OPG in both oral and periodontal pocket epithelium [36]. Moreover, several lines of evidence reveal that RANKL and OPG levels have the tendency to the reduction and elevation upon non-surgical periodontal treatment, respectively [37, 38].

As mentioned previous, miR-146a down-regulates IRAK-1 and TRAF 6, which are the key adaptors in signaling by toll like receptors (TLRs) and cytokine receptors [4]. Down-regulation of these adaptor proteins result in the abolished activation of nuclear factor-kappa B, which is a central transcription factor in the transcription of pro-inflammatory genes [39]. Other studies have demonstrated that pro-inflammatory cytokines stimulate the expression of RANKL and suppress the production of OPG [17–19]. These studies suggest that the reduced level of RANKL and increased expression of OPG during disease progression may relate to the elevated level of miR-146a in the early disease stage to exert a negative impact on pro-inflammatory cytokine productions and thereby reduces disease progression.

In the next step, to deremine the possible impact of miR-146a on the levels of RANKL and OPG, the correlations of miR-146a level with these molecular factors were evaluated. Our results showed that miR-146a level was not correlated to RANKL and OPG levels. Another result of the current study indicated no significant relationship between miR-146a level and OPG/RANKL ratio, which was a further confirmation in regard to no correlations of miR-146a with RANKL and OPG levels after non-surgical periodontal therapy.

## Conclusions

Although it is thought that miR-146a has indirectly effects on RANKL/OPG axis through reducing pro-inflammatory cytokines. The results of this study provide evidence to indicate that the increased level of miR-146a is not associated with RANKL and OPG expressions and RANKL/OPG ratio in periodontal tissue after non-surgical treatment. Nevertheless, additional studies with larger sample sizes are required to confirm our findings and explain the possible mechanism(s) involved in anti-inflammatory effects of miR-146a on disease development in patients without initial periodontal therapy. It is worthy that future studies will be designed to clarify the impacts of miR-146a on other mediators such as prostaglandins, leukotrienes, macrophage colony-stimulating factor, galcectin-3, NLRP3, transglutaminases, and fibroblast growth factor, which play indispensable roles in bone homeostasis and periodontal disease progression [6, 7, 40].

## Data Availability

All data generated or analyzed during this study are included in this published article. No sequencing data generated in this study to deposit in a suitable public repository such as the National Center for Biotechnology Information (NCBI) database. The raw data are available from the authors to any researcher who wishes to collaborate with us. Correspondence should be addressed to Hossein Motedayyen at the following email address. Email address: hmotedayyen@gmail.com. Specific primers were designed using Allele ID 7.5 software (Premier Biosoft) and checked by Primer-BLAST (NCBI) which is an open access database.

## Abbreviations

TLR: Toll like receptor
MiR-146a: MicroRNA-146a
PD: Probing depth
CAL: Clinical attachment loss
MiRNA: microRNAs
RNA: Small ribonucleic acids
RANKL: Receptor activator of nuclear factor-kB ligand
OPG: Osteoprotegrin
TNF-α: Tumor necrosis factor-alpha
IL-1β: Interleukin-1β
IL-6: Interleukin-6
cDNA: Complementary DNA
Real-time PCR: Real-time polymerase chain reaction
GAPDH: Glyceraldehyde 3 phosphate dehydrogenase
SD: Standard deviation
IRAK-1: IL-1 receptor-associated kinase-1
TRAF 6: Tumor necrosis factor receptor-associated factor 6
